# HeriLedger—A New Generation of Blockchains for Cultural Heritage Preservation

**DOI:** 10.3390/s22228913

**Published:** 2022-11-18

**Authors:** Denis Trček

**Affiliations:** Faculty of Computer and Information Science, University of Ljubljana, Večna pot 113, 1000 Ljubljana, Slovenia; denis.trcek@fri.uni-lj.si

**Keywords:** distributed ledgers, internet of things, sustainability, quantum computing resistance, privacy, cultural heritage, business models

## Abstract

The immense success of Bitcoin has also highlighted the hidden potential of blockchains and distributed ledgers in general. However, most blockchains are based on the so-called Proof-of-Work principle, which requires significant resources, making them unsuitable for the growing number of Internet of Things devices, not to mention other problems such as ensuring privacy and resistance to quantum computing. This paper, therefore, analyses current approaches in the field of ledgers with applications for the Internet of Things. Based on this, it presents a new ledger architecture for cultural heritage preservation that is energy sustainable and tailored to smartphones while pushing the deployment boundaries closer to the rest of the Internet of Things world. It is also resistant to quantum computing and provides privacy with accountability. Moreover, the developed solution considers not only the core technological structure but also its broader social (business) context, i.e., links to the tourism sector. This is performed by incorporating the relevant business model, as business models are important for the successful adoption of new technologies.

## 1. Introduction

The invention of Bitcoin (BC) started a cryptocurrency revolution in 2009 that resulted in over 10.000 cryptocurrencies today [1]. Due to its reliance on a blockchain infrastructure, BC led to another kind of revolution—ledger-based applications that promise to become at least as important as Bitcoin itself [2]. The main reason is that ledgers offer, for the first time, a solution to an inherent problem of digital currencies, namely the problem of double spending. In fact, blockchain technology enabled the introduction of the concept of “original and copy” in the digital world. Moreover, it enabled the integrity of stored objects and also verifiable links to current and previous owners, which in turn enabled new services such as non-fungible tokens (NFTs) [3].

However, there are some important drawbacks of the traditional blockchain architectures (TBC) mentioned above. The first is energy consumption. TBC uses the Proof-of-Work (PoW) principle, where a block of transactions is hashed along with the hash of the previous block and the changing nonce to obtain a certain number of leading zeros when calculating the hash of all these elements. This is of increasing concern as global BC mining consumption has already exceeded the total electricity consumption of economies such as Switzerland in 2019 [4]. It has therefore been banned in many countries, including China, followed by Kazakhstan and others [5]. On the other hand, the Internet of Things (IoT) devices are becoming the dominating part of the computing population in the global network, which is estimated to already exceed 10 billion [6]. However, IoT devices have many specifics, most notably limited computing resources and constrained energy availability—and IOTA is an alternative architecture that is supposed to suit IoT [7]. It is still in its early stages of development, which also holds true to some extent for TBC [8].

Strongly related to these specifics are wider social (business) requirements and consequences. Focusing on a particular technology alone is often not sufficient for its successful deployment without addressing the business context [9]. BC is clearly a techno-social system, where the social part has been remarkably well addressed by its inventor. In the case of BC, this business context is about the prevention of crises caused by unacceptable practices of the banking industry and an idea to “free” the citizens from being “slaves” of (central) banks, while the limited number of BCs should prevent devaluation pressures, etc. Last but not least, BC is a native payment system for the digital world.

Ledgers (often used to support digital payment implementations) have been adopted in many sectors to date. However, surprisingly, there are almost no such implementations for cultural heritage preservation. One exception is the Chinese implementation of a testbed solution for the murals in the Mogao Caves (UNESCO heritage), which uses a private blockchain [10]. However, it is a closed system, and it does not use cryptography, which is the case in the rest of the blockchain world, as the Chinese government has recently banned the use of unregulated cryptography.

Therefore, this paper presents a new approach to ledger technologies for IoT devices (especially cell phones) intended for cultural heritage preservation. It is sustainable, resistant to quantum computing, provides privacy with accountability, and considers the mentioned business context also with an appropriate reward system. Further, it is an open solution that uses strong cryptography.

**Figure 1:**
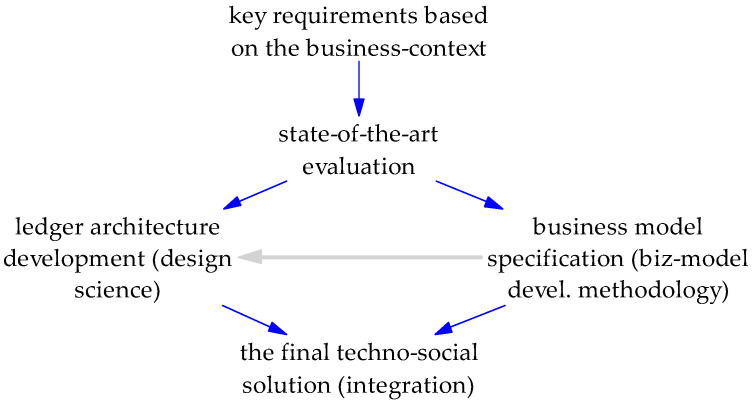
The top-level methodological approach of this paper.

The top-level methodological research approach is given in Figure 1., while the above-specified motivation elements lead to the following research questions, addressed in this paper:

1. Which technology, TBC or IOTA, better meets the heritage preservation needs when considering mobile phones (and IoT), along with sustainability, privacy with accountability, and quantum computing resistance? Can this be a variation of one of them or some hybrid solution?
2. As current approaches often pay little attention to a fine-grained business context—what are the needs of the intended users and organizations in the given cultural heritage context?
3. Apart from cryptocurrency, can there be another kind (or a variation) of users’ incentive for linking the technological and business domains?
4. Is there a structured approach for appropriately linking the technological and business domains through business models, which can serve as a reference approach?

The core methodology deployed is design science. Its objective is knowledge development that the professionals of a particular discipline can use for designing solutions to solve their problems [11]. In the case of IT, these are the elements of design science research [12]: A technological solution must be relevant to a business problem (problem relevance), which is a starting point for appropriate model construction, method, or an instantiation of technological solution (artifact design). The latter step depends on finding an effective solution that utilizes available means while considering the laws of the problem domain (design as a search process). Next, the quality, utility, or efficacy of the developed solution must be based on rigorous design and evaluation (research rigor and design evaluation). Further, verifiable contributions must be clearly stated (research contributions). Finally, these elements need to be effectively presented to technology and management audiences (communication of research).

The rest of the paper is organized as follows. In section two, the state of the art is given together with its analysis. This is followed by the methodological approach details in the third section, which is the basis for a new solution presented in the same section. There is a discussion in the fourth section and conclusions in the fifth section. The paper ends with acknowledgments, funding details, a conflict of interest statement, and a bibliography.

## 2. State-of-the-Art Overview and Evaluation

Blockchains are representatives of distributed ledger technologies (DLTs) for storing and sharing data in distributed, consensus-based environments without a central administrator. The data are stored in an immutable way that ensures their integrity in a transparent manner, with changes being traceable.

There are currently two main DLT architectures: TBC and IOTA [13]. The latter ledger is mainly analyzed in this section for two reasons: First, TBC details are well-known and understood (see, e.g., [14]), while IOTA is less understood. Second, the state-of-the-art overview in this field reveals that IOTA is extensively evaluated against TBC. Therefore, focusing on IOTA also gives an evaluation against TBC.

**Figure 2:**
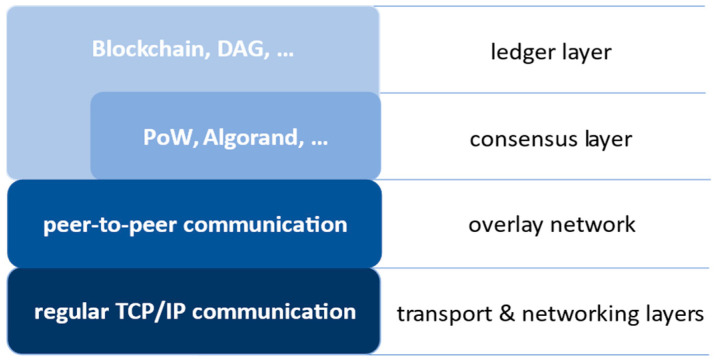
The core networking with overlay and consensus protocols for ledger technologies.

Starting with the DLTs networking, the basis is TCP/IP model [15] (see Figure 2). Next comes peer-to-peer (P2P) networking with its own protocols for bootstrapping, storing, and finding the content (distributed hash tables usually serve this purpose [16]). P2P networks can be established ad hoc, there is no need for centralized entities, e.g., specialized servers, and they are storing the content in a redundant way among the participating nodes. Therefore, the more nodes there are, the more resilient the structure is against the loss of stored content or malfunctioning of a certain node, which is essential for the accessibility and integrity of the stored data.

P2P networking also introduces certain disadvantages, such as distributed file synchronization issues. Further, hosts’ performance may be negatively affected, especially if P2P architecture requires a “fair” load share, i.e., when during downloading, one is also forced to upload content. Furthermore, security issues may be notable, where the Sybil attack remains an inherent problem. Further, users’ privacy may be endangered (with a forced upload, one may involuntarily take part in illegal content sharing). Further, network malfunctioning can affect the consensus protocol—messages may arrive too late or not arrive at all.

Focusing now on IOTA—it addresses not only the specifics of IoT devices but also compensates for some drawbacks of TBC, which are energy consumption, latency, scalability problems, and transaction costs [7,17]. In order to counter these problems, a new validation mechanism is introduced, where IOTA classifies nodes into full and light ones. The light ones with low computing resources rely on full nodes’ support for validations when attaching new transactions to the tangle. As load balancing may be a problem, a mechanism for a fair distribution of workload among full nodes is needed [7]. Consequently, IOTA has a solid, scalable transaction confirmation capability, which improves BC’s inefficiency and unsustainability by eliminating the mining pools [18]. Nevertheless, it may still be challenging to derive reliable performance guarantees.

While TBC is a chain of blocks, IOTA is a directed acyclic graph, DAG (also called the tangle). The unvalidated transactions in IOTA are referred to as tips, and tip selection is performed using an asynchronous, stochastic approach, the Markov chain Monte-Carlo algorithm [19]. However, this stochastic approach may lead to a parasite chain attack that disrupts the immutability and irreversibility of the ledger. Put another way, DAG ledgers with stochastic attachment mechanisms do have better scalability and faster transactions compared to TBC. However, the protection against double-spending may lead to an unstable system where some transactions are not validated (these are called orphaned transactions). To prevent this, an improvement of the attachment mechanism has been proposed that ensures the validation of all transactions in a finite time [20]. To further improve scalability, an approach in [21] deploys a “micro-payment enabled over the top” streaming platform based on the “pay-as-you-go” and “consumption based” models.

IOTA is supposed to provide a promising basis for IoT devices, but when these devices generate transactions and micropayments, using fees to manage a network is not optimal due to the lack of resources. Consequently, a feeless IOTA architecture can be an answer to this problem. However, the lack of fees can be exploited, and malicious nodes can generate numerous transactions that will lead to denial of service (DoS) attacks. In [22], verifiable delay functions are proposed to counter this problem because they are non-parallelizable, hard to compute, and easy to verify.

As to IOTA security, privacy and trust, these issues are analyzed extensively in [17]. Although IOTA is supposed to offer resilient security and trust mechanisms, the mentioned study presents many vulnerabilities. Security is further addressed in [23]—the survey identifies general threats and attack vectors while presenting solutions for remediation of the compromised security, as well as methods for risk mitigation, with prevention and improvement suggestions. Regarding privacy, this is addressed in [24] (where privacy is interchangeably used with anonymity). However, in fact, IOTA makes no notable advances when it comes to privacy because the proposed enhancements are based on the off-ledger mixers paradigm, already known in the BC world.

Finally, a different and good overview of TBC’s suitability for IoT is given in [25]. The paper shows how IoT with TBC facilitates the sharing of services and resources, thus creating a marketplace of services between devices and allowing workflow automation in a cryptographically verifiable way. It should be emphasized that the paper is in favor of TBC-IoT coupling through smart contracts, and this coupling is expected to cause significant transformations across industries while paving the way for new business models.

## 3. The Methodological Approach to HeriLedger Development

The methods needed to address the research questions, stated at the beginning of this paper, include the following elements:

- Design science as the basis, complemented with,
- Risk management,
- Cryptographic protocols development,
- And business model development.

**Figure 3:**
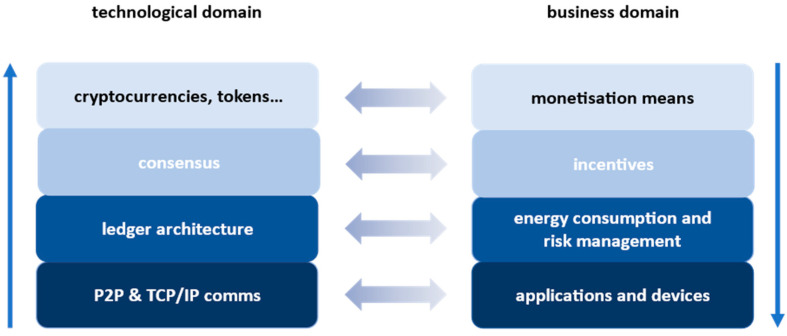
Llinking the technological model (on the left) with the business model (on the right) and the corresponding layers.

These methods will be used in a procedural approach that consists of two aligned stacks given in Figure 3. The approach starts in the business domain with a business model outline having the following main elements:

- The goal of the model is to enable the preservation of digital and digitized cultural heritage in an open and immutable way with DLTs based on cryptography.
- The solution should link the main players, which are cultural heritage institutions (like museums, galleries, and archeological sites), with the main users, which are domestic and foreign tourists and education institutions. By doing so, it should reduce preservation costs for cultural institutions while providing tangible benefits to the users.
- The solution should enable adding value by, e.g., enabling the use of the artifacts preserved in a DLT in new ways such as mixed reality, virtual reality, etc.
- The main purpose of the developed DLT is the preservation of the artifacts and not transactions with them—although these may happen, their volume can be neglected (e.g., a transfer of ownership in case of donations or a relocation from a gallery into a museum, etc.).
- The solution should meet the technological reality—by focusing on smartphones, even undeveloped countries can benefit from the solution (note that according to [26], 83.72% of the world population owns a smartphone).

The above-described main elements present the core. A more detailed elaboration, as required by the methodology given in [27], should also include key activities, key resources and partnerships, channels, etc.—and this follows in the rest of the paper.

### 3.1. The Business Context Elaboration

The business context addressed is cultural heritage preservation linked to the tourism sector. Cultural heritage, as stated, refers to tangible and non-tangible artifacts, which are digitized, or natively digital, conceived in the digital domain. To properly engineer the new DLT’s integrity and privacy support vs. users’ incentives for its maintenance, risk management has to be performed. It starts with threats in relation to resources’ vulnerabilities. Based on the resources’ value and the probability of a threat successfully leading to an attack, risk estimate is obtained. In this paper, risk management will be performed informally—a detailed view of related issues with an archetype model can be found in, e.g., [28].

The first question is—how much is cultural heritage worth? This is rather hard to judge, as heritage is very diverse, ranging from fine arts works from world-class painters to folklore music, where the authors are unknown. Thus, on one hand of the spectrum, there is a strong market potential behind it, while on the other part of the spectrum, it can hardly be subject to market principles. A reasonable assumption is that also the first kind of cultural heritage is owned by a community or society through its institutions, such as museums or galleries. This effectively removes a piece of fine art from the market. Thus, although cultural heritage is of highest importance to nations, ethnic groups, and other communities, it is assumed it is not subject to free market principles and can be considered a public good.

This is the basis for appropriate incentives for users who engage in a DLT maintenance process that preserves heritage. Traditional mining is not appropriate, as no financial rewards such as BCs should be granted. First, non-cash options for incentives are needed due to the low market value of mined artifacts. These include e-checks, e-tokens (also called e-vouchers), or e-reward points (e-coupons). In order to minimize similarities with traditional payment systems and reduce associated risks, e-checks and e-tokens are excluded. They may lead to secondary trading and exchange due to their transferability. The preferred choice, therefore, remains e-reward points, which can only be spent once and can only be assigned to a specific user based on his (her) participation in the operation of the DLT. The points can be exchanged for tangible or intangible goods ranging from museum passes to tickets to cultural events to exhibition catalogs. Consequently, the interest of attackers is low, as the estimated risks for each participant are in the range of a few dozen or a few hundred EUR per year.

### 3.2. Technological Solution Development

**Figure 4:**
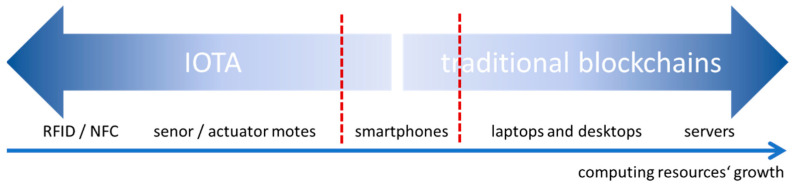
Positioning smartphones with respect to their computing resources for supporting DLTs.

The core computing devices for the desired DLT are smartphones (they inherently belong to the IoT world as it is rather straightforward to turn them into IoT devices [29]). Their computing power is in the middle range of the main contemporary computing devices when computing resources are considered (see Figure 4).

Therefore, mobile phones can be considered the strongest representatives in the IoT world (thus, IOTA architecture may be a borderline platform), while in relation to the TBC, they are among weaker representatives (being a borderline platform again). Nevertheless, they are the right fit for our purposes because of the business context requirements. Further, the risk analysis excludes traditional cryptocurrency mechanisms, while cultural artifacts are expected to be “burnt into” the new ledger “once and for ever” (very few transactions are expected besides the initial one)—this reduces the computing resources demands. However, “once and forever” functionality also implies quantum computing resistance that TBC does not provide, while IOTA did, but this latter functionality of IOTA has been abandoned recently [30]. On top of this, none of these ledgers provides privacy with accountability, which can be provided with mobile phones. Thus, the logical question now is which of the mentioned DLTs should be chosen? And can it be used in its pure form or in an adapted or hybrid form?

Before giving an answer, consensus protocols must be addressed. TBC and IOTA both depend on proof of work (PoW), which leads to sustainability problems. Alternatives with a lower carbon footprint include proof of stake (PoS), where transactions are confirmed by those with most interest in its correctness, proof of authority (PoA), where transactions are confirmed by a trusted authority, and proof of burn (PoB), where miners permanently eliminate crypto coins and in proportion to the “burnt” coins they are granted to write blocks to the chain (some coins are spent to attain associated rewards, which may be coins again). However, these alternatives do have downsides: PoS and PoB could lead to a “rich getting richer” situation because they reward users possessing lots of coins, while PoA eliminates the core tenets of DLTs, as it gives the power into the hands of one central authority, which may also become the single point of failure [31].

From the game-theoretic point of view, many of the protocols are not yet well understood, not to mention that they have not been tested extensively on a large scale, which is not the case with PoW. Although IOTA could be a candidate for the heritage DLT, it has additional weaknesses besides the presented ones: it is implemented for approximately five years, while BC has been around for almost fifteen years—and BC has been tested on a much wider scale than IOTA.

Summing up, as TBC is an industry standard, which is widely studied, there is a strong reason to choose it for the basis. However, its PoW must be adjusted to make the solution sustainable and appropriate for the business context. This can be achieved by replacing the traditional public key cryptography solutions for signing and using only strong one-way hash functions (OHFs). The power consumption of most OHFs (like SHA256 and SHA512) for various sizes of input files is notably lower when compared to symmetric and asymmetric algorithms [32]. Thus, OHFs present an appropriate substitute for TBC security primitives if a signature scheme can be implemented by using them—which turns out to be the case with, e.g., Lamport’s scheme [33]. Further, by using OHFs with a lower number of output bits (in line with risk analysis) or by reducing the number of required leading zero-positions when mining the blocks of transactions, the energy consumption can be further adapted.

Although Lamport’s scheme has some limitations (each private and public key pair can be used only once), this is not a serious issue in our case, as the signing is needed only once because a transaction that includes a heritage artifact into a ledger is of a “once and for all times nature”. Being completely based on OHFs, Lamport’s scheme also provides quantum computing resistance (this latter property is ensured, e.g., by Merkle–Damgård architecture [34], where the operations cannot be parallelized but must be executed sequentially).

Lamport’s scheme goes as follows (OHF is assumed to produce 256 long hashes):

1. A signer generates a secret key *sk*, which consists of two sequences of 256 b long random values:

$$sk_{0}=sk_{0,1},sk_{0,2},\ldots ,\;sk_{0,256}\;sk_{1}=sk_{1,1},sk_{1,2},\ldots ,\;sk_{1,256}$$

2. Next, the signer generates the corresponding public key *pk* that also consists of two sequences, each 256 b long, which are obtained by hashing the secret key values. The obtained sequences are made publicly available, and these represent the signer’s public key:

$$pk_{0}=H\left(sk_{0,1}\right),H(sk_{0,2}),\ldots ,\;H(sk_{0,256})\;pk_{1}=H(sk_{1,1}),H(sk_{1,2}),\ldots ,\;H(sk_{1,256})$$

3. When signing a file F, the signer first hashes the file and looks at the i-th bit in the hash—if this bit equals 0, the signature value is *sk*_0,I_; otherwise, it is *sk*_1,i_. Now when any third party checks the signature, (s)he hashes the i-th position in the signature of F. If it equals 0, the check is made with H(*sk*_0,i_) (if its value is *pk*_0,i_); for value 1 at the i-th position, *pk*_1,i_ is compared with H(*sk*_1,i_).

It is obvious that the longer the hash of the original message, the less likely its sequence of bits is falsified by an attacker (by relying on the birthday paradox [28]). Further, the signature is resistant to quantum computing as opposed to TBC, which deploys discrete log-based elliptic curves cryptography [34]. It should also be noted that the solution is by no means limited to 256 bits—what is needed is OHF with preferably adjustable length of hashed outputs (a good overview of such functions can be found in [35]).

Now a new extension for the above signs can be introduced. It will be referred to as Backwards Chained Keys Scheme (BCKS), and it goes as follows (the subscripts denote the key’s horizontal sequence number and its role, whether it is for signing for a bit with value 0 or 1, while the superscripts denote the key-pairs’ vertical sequence number):

1. A signer generates the initial (parent) public and secret key pair:

$$sk_{0}^{0}=sk_{0,1}^{0},sk_{0,2}^{0},\ldots ,sk_{0,256}^{0}\;sk_{1}^{0}=sk_{1,1}^{0},sk_{1,2}^{0},\ldots ,sk_{1,256}^{0}$$

$$pk_{0}^{0}=H(sk_{0,1}^{0}),H(sk_{0,2}^{0}),\ldots ,H(sk_{0,256}^{0})\;pk_{1}^{0}=H(sk_{1,1}^{0}),H(sk_{1,2}^{0}),\ldots ,H(sk_{1,256}^{0})$$

2. Afterward, s/he generates *n* (child) secret keys and stores the initial {$sk_{0}^{0}$,$\;sk_{1}^{0}$} pair:

$$sk_{0}^{1}=H(sk_{0}^{0})\;sk_{1}^{1}=H(sk_{1}^{0})$$

$$sk_{0}^{2}=H(sk_{0}^{1})\;sk_{1}^{2}=H(sk_{1}^{1})\;\ldots \;sk_{0}^{n}=H(sk_{0}^{n-1})\;sk_{1}^{n}=H(sk_{1}^{n-1})$$

3. Next, by hashing the private keys’ values, given in step 2 (s)he obtains the corresponding (child) public keys.

4. Finally, the signer uses these public-private key pairs in reverse order, making the first signature with
  $$pk_{0}^{n}=H(sk_{0}^{n})\;pk_{1}^{n}=H(sk_{1}^{n})$$
  Then with $\left\{pk_{0}^{n-1},pk_{1}^{n-1}\right\}$, and so on in the corresponding reverse manner the private keys are used.

BCKS provides similar functionality to the so-called HD wallets in BC environment, which can derive child private keys and child public keys from their parent private keys and parent public keys based on ECC [36,37]. However, as opposed to these wallets, BCKS is quantum computing resistant. It also supports privacy with accountability property, elaborated below. Finally, why are such sequences needed if we consider only one transaction per e-coupon? Clearly, one user may have multiple such “one-shot” transactions because through mining (s)he may be awarded more than just one e-coupon. Further, if a public key is used only once, as enabled by the BCKS chain, an identity is undisclosed (note that there are no certificates). However, we also want to provide accountability for the misbehaving parties. This requires appropriate modification of the TBC public addresses, where e-coupons are linked to. In this case, TBC address is not just a hashed public key of the winning miner but a concatenation of a zero-knowledge proof (ZKP) element and a public key, where ZKP is based on the user’s mobile phone number.

Let *mpn* stand for user’s mobile phone number (which represents an integer), *pk* for his/her Lamport’s public key, *sk* for the corresponding private key, *H* again for a strong one-way hash function, “||” for concatenation, *M* for the successful miner identity, and *I* for a cultural institution. The protocol called Privacy with Linkable-ID (PwLID), which enables accountability goes as follows:

1. *M* authenticates his/her mobile phone to the wallet via *mph*. The wallet generates a challenge that is sent via mobile network to the phone. The user enters the challenge, which activates the wallet.
2. *M*’s wallet calculates *H*(*pk^n^* || *mpn*) = *pa*, where *pa* stands for *M*’s linkable public address, and announces this public address (in ordinary TBC, public address is only a hashed public key value, extended with a checksum value, which can be also added in the case of PwLID). *M*’s wallet discards the *mpn*.
3. When successful, the network awards *M* with an e-coupon by digitally signing a transaction of this e-coupon to *pa*.
4. When *M* wants to spend the e-coupon, (s)he does so at *I* by making a transaction with her/his private key *sk^n^* to the institution’s (“sink”) public address.
5. To prevent forgery, the institution *I* requires her/his *pk^n^* to check the signature.
6. Next, the institution *I* starts the ZKP to verify that *M* knows the secret behind *pa*, i.e., his/her *mpn* (so that the right mobile phone number is awarded, the one that has spent computing resources). ZKP is such that it is quantum computing resistant, e.g., [38].
7. If there is a reason to break privacy (accountability is required), *I* also requires the *mpn* to fully check the transaction and indirectly identify *M* before accepting the e-coupon.

Note that *mpn* is not enough for full identification of an individual (also, cultural organizations do not have such rights). However, it is sufficient for legal procedures because the institution can hand *mpn* over to official authorities, which can require the disclosure of the owner’s identity from a mobile network operator.

**Figure 5:**
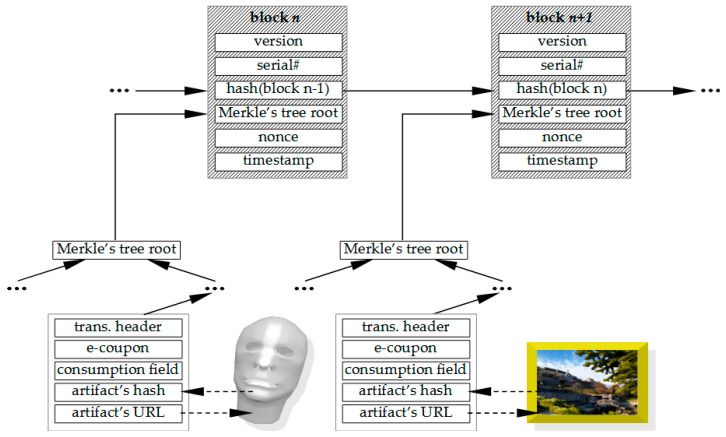
HeriLedger blocks structure details.

The transaction details can now be given (see Figure 5). The block header structure is as follows:

- Version of the software/protocol,
- The serial number *SN* of the transaction block,
- The hash of the previous block (except for the first block),
- The transactions’ Merkle’s tree root,
- The nonce *N* of the block that has led to “solution” of the block that is being mined,
- The timestamp when the block was approved (obtained from a mobile network).

The transactions’ core consists of Merkle tree and has the following elements:

- The transaction header with a serial number in the form *SN.M*, where *SN* denotes a block’s serial number, and *M* denotes transaction’s serial number within the block;
- The awarded e-coupon with the transaction’s block serial number (each coupon has a fixed value) linked to a *pa*;
- The spending of the e-coupon, one for each e-coupon issued (in case of unspent e-coupon, this value is “null”, otherwise this field has the owner’s signature of the consumed e-coupon to a “sink address” of an institution where the e-coupon was exchanged for a tangible product or a service);
- The hash value of the (preserved) cultural artifact;
- The URL pointing to the location where the mentioned digital/digitized artifact is stored (this field can also be used as a cross-ledger link, e.g., an URL pointer to an NFT ledger).

These elements have the corresponding scripts that are necessary to execute the operations of hashing and OHFs-based signing. As to the rest of the architecture, it remains compliant with the TBC, and its details can be found in, e.g., [37].

### 3.3. Business Model Specification

The initial elements of the needed business model were given at the beginning of this section. However, the required business model needs further elaboration on its elements.

First and foremost, cultural heritage institutions are typically not-for-profit private and public (community or state-funded) organizations. They are often run with limited funds, so their business processes’ optimization is very much desired. The proposed solution provides some important support in this respect. It enables outsourcing of the preservation processes to tourists via a mobile application.

Next, as described in [27], a business model addresses how an organization (or an industry segment) creates, captures, and delivers value to its customers. This is achieved through the following building blocks: customer segments, value proposition, key activities, key resources, channels, customer relationships, key partnerships, cost structure, and revenue streams. Putting these elements into the context of HeriLedger solution, the core specification of the mentioned elements is as follows:

(a) Customer segments include domestic and foreign tourists, where the meaning of tourists has to be interpreted rather broadly to include visitors to galleries, cultural events, attendees, etc. It is common for all of them that they provide free computational resources on their mobile phones for HeriLedger.
(b) Value proposition is manyfold. Regarding cultural heritage institutions, the value proposition is to reduce their operations’ costs by outsourcing the heritage preservation processes. Regarding the tourists, these become a part of something of greater importance, while the services offered can be the basis for additional added value, e.g., in enhanced or virtual reality applications. This latter case is especially appropriate for educating children and younger generations. As to society in general, this solution enables wider, even global, accessibility to cultural heritage.
(c) Key activities are certainly documentation and preservation of cultural artifacts, which, if not already digital, have to be digitized. Further, the central key activity is the initial production of the HeriLedger application, while its maintenance is not expected to require notable inputs. Due to its nature, this app could be open source, initially funded by a national government or EU funds. Finally, appropriate coordination with tourist organizations is needed.
(d) Key partnerships are mainly those related to tourist organizations. Clear interests of tourist organizations must be identified. The direct incomes are straightforward to identify, but the proposed solution contributes to the incomes indirectly by increasing tourist visits. This leads to higher incomes in this sector and gross domestic product in general.
(e) Key resources are digitized and digital cultural heritage artifacts, tourists, and the mobile app for bootstrapping and maintaining the new ledger. It is necessary to emphasize that the related APIs will make it an open solution and enable its use by other services, such as augmented and virtual reality.
(f) Costs’ structure includes core processes costs and their migration to the mobile app development and maintenance, and marketing of the new ledger service until it is accepted, i.e., up and running. However, if open-source implementation is provided, the latter costs will be negligible for cultural institutions.
(g) Customer relationships—these cover primarily tourists with their involvement in the heritage preservation processes through e-coupons, being traded for underutilized mobile phones and computing resources.
(h) Channels—the main one is the so far weak (or non-existing link) between the tourist sector and the heritage preservation sector. This will be another important improvement enabled by HeriLedger solution.
(i) Revenue streams are directly identifiable by heritage organizations and institutions, while for tourist organizations, they are, as mentioned, mainly indirect. This will likely require certain state involvement to promote the presented approach until its benefits become visible to a larger society.

The above elaboration of the business model elements should be sufficient, and their further, detailed elaboration will be dictated by a particular business environment.

## 4. Discussion

In this section, the resource consumption analysis is given, preceded by an additional analysis of the threats to the developed system’s security and privacy. It covers:

- Sybil attack and general security,
- e-coupons stealing and their double spending,
- privacy provisioning,
- quantum computing resistance and,
- lightweight implementation.

A generic attack in TBC environments is a Sybil attack, where false nodes pretend to be legitimate ones and try to take over the consensus process. The PoW prevents this threat to a large extent, but the BC variant of PoW leads to unsustainability. In HeriLedger’s case, the attackers are considerably less motivated for such kinds of attacks despite somewhat lower computational demands. The core reason, as stated in the risk analysis, is that the reward for successful mining is limited to a few hundred EUR per year per participant. Further, the reward is not a traditional currency and is non-transferable. Moreover, all parties, including potential attackers, are linkable to their true identities through the PwLID protocol (which deploys hashed structures with mobile phone numbers). Thus, the potential attackers can be caught, e.g., at the point of cashing out the e-coupons.

Double spending on e-coupons is inherently prevented, as a user’s only transaction happens when this coupon is bound to the sink address at the cultural institution (while the institution may make further transfers to, e.g., the national ministry’s sink address). Regarding the stealing of e-coupons, this is prevented by strong encryption, just like in the TBC case. Furthermore, the general security provisioning remains the same, with the main distinction being the use of crypto mechanisms based solely on OHFs, which are quantum computing resistant.

Further, privacy is provided with the PwLID protocol that deploys ZKP, but with a notable improvement—privacy can be broken if needed because of the links to a SIM or mobile phone number. Therefore, misbehaving entities can be identified.

**Figure 6:**
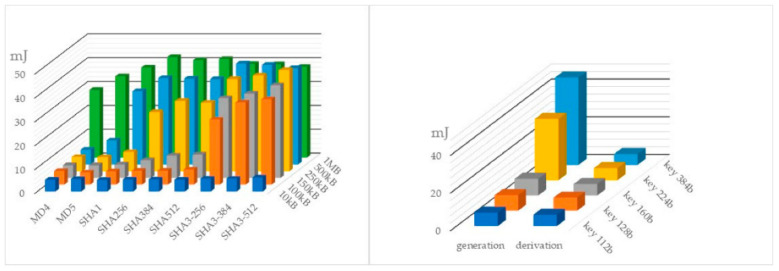
The energy consumption of the main crypto mechanisms in relation to their type, key length and file size (hash functions on the left, and ECC-Secpr1 on the right).

Finally, the scheme is lightweight. Using Lamport’s scheme, 512 hashes are needed for private key derivation, 512 hashes for public key derivation, and 512 hashes for signature verification. This means 1024 hashes for computing the keys and 512 hashes for signature verification (all with 256 b long values). Assuming SHA-256 with 5.06 mJ consumption for one hashing of a file smaller than 10 kB, the total consumption for computing both keys is 5184.44 mJ, and for signature verification, 2590.72 mJ (energy consumption of related cryptographic mechanisms is given in Figure 6 [32]).

This somewhat exceeds ECC consumption in TBC. However, ECC is not quantum computing resistant. Further, ECC in TBC is needed for an “endless” sequence of transactions, while in the case of HeriLedger, the number of transactions per key pair is negligible. In addition, Lamport’s scheme can be further optimized by signing only, e.g., “1” bit-values, and adding a checksum, which is an integer that denotes the number of “1” bit-values. This checksum also has to be signed. By doing so, the total number of bits needed for the signature can be approximately halved on average.

Next, the ledger size—as of July 2022, the BC blockchain size was approx. 400 GB (https://www.statista.com/statistics/647523/worldwide-bitcoin-blockchain-size/, accessed on 10 November 2022). Considering now a national heritage ledger, Table 1 gives the number of artifacts in Slovene museums (the latest figures are from the year 2010 [39]):

There are 6,560,000 artifacts. Each digital signature requires 32B for the hash of an artifact and 32B*256 for its signing, a total 8 KB, and by using the mentioned checksum, this is reduced to 4 KB. Signing all the artifacts for the ledger requires, therefore, 6.56 × 10^6^ × 4 KB, which is approx. 26 GB (other fields in the ledger are neglected because they are notably smaller than the signatures). Further, the number of transactions in the BC network is 230,000 per day (as of November 2021) (https://www.statista.com/statistics/730838/number-of-daily-cryptocurrency-transactions-by-type/, accessed on 10 November 2022), which means approx. 83.95 million transactions per year. The number of initial transactions for the national HeriLedger, as mentioned, is 6.56 × 10^6^, and then it is assumed that new artifacts will appear at a much lower rate, e.g., 10^4^ per year. This means that in the case of Slovenia, the national ledger with approx. 27 MB to 28 MB would be currently sufficient.

The comparison of both ledgers is given in Table 2, where the rest of the properties are not evaluated because both solutions are otherwise architecturally aligned. Evidently, the HeriLedger architecture fulfills the performance expectations and provides the desired properties.

## 5. Conclusions

This paper presents a novel ledger solution called HeriLedger that focuses on cultural heritage preservation and is linked to the tourism segment. Tourism and cultural heritage are organic twins, and so far, there are almost no such blockchain-based or other distributed ledger-based implementations. One exception is the aforementioned Chinese implementation of a testbed solution for the murals of the UNESCO World Heritage Site Mogao Caves. However, this implementation uses a private blockchain owned by the giant company Tencent. It also does not use cryptography due to recent Chinese government regulations, as is the case in the rest of the blockchain world.

The ledger in this paper offers many important improvements. Unlike traditional ledgers based on the BC architecture, it is sustainable in terms of computational resource consumption. Second, it is also resistant to quantum computing. Third, it provides privacy with optional accountability. Fourth, it is developed in parallel with the business context, and the core business model associated with this solution is presented.

Since we want to provide a solution for the IoT world in general, any step to cover a larger part of the IoT population is high on the wish list. By focusing on cell phones for (business) reasons, we achieve an indirect gain in this regard—porting the presented application to cell phones is quite easy, as cell phones are also an important part of the IoT world [29,40].

Finally, IOTA was considered the first option in this paper because it is designed for the IoT world. However, the analysis presented shows that a modified TBC is the better option for many reasons, including the fact that it has been around for a long time, and we are, therefore, familiar with its weaknesses. IOTA, in contrast, has only been deployed for a few years and its many potential vulnerabilities have yet to be discovered.

So instead of taking a revolutionary path, the presented approach opts for an evolutionary path by using a proven solution that is appropriately improved and adapted to business needs. By doing so, the way is paved for Heritage Preservation 4.0.

## Figures and Tables

**Table 1:** Numbers of cultural heritage artifacts in Slovene museums.

archeology	3,800,000
history	1,700,000
ethnology	150,000
fine arts and crafts	400,000
geology and natural history	300,000
science and technology	10,000
other	200,000
**TOTAL**	**6,560,000**

**Table 2:** Comparison of TBC and Heriledger (for a signature and its verification with ECC-160b one order of magnitude lower energy consumption is assumed than for RSA).

	Computing Keys (mJ)	Signing (mJ)	Verification (mJ)	Number of Transactions/yr	Energy Costs/yr
TBC	60 (160 b)	4.6 (160 b)	0.7 (160 b)	83,950,000	∞
HeriLedger	5184	~0	2591	10,000	5184 mJ × 10,000 = 51,840 J
	current ledger size	Ledger growth/yr	quantum. comp. resistance	privacy with accountability	the rest of properties
TBC	400 GB	~60 GB	NO	NO	ND (no difference)
HeriLedger	27 MB	10,000 × 4 KB ≈ 40 KB	YES	YES	ND (no difference)

(As stated, TBC energy consumption exceeds those of economies such as Switzerland).

## Data Availability

Not applicable.
